# Immunopathological comparison of *in ovo* and post-hatch vaccination techniques for infectious bursal disease vaccine in layer chicks

**DOI:** 10.3389/fvets.2022.947522

**Published:** 2022-07-26

**Authors:** Iqra Zaheer, Wei Chen, Ahrar Khan, Abdelmotaleb Elokil, Muhammad Kashif Saleemi, Tean Zaheer, Muhammad Zargham Khan

**Affiliations:** ^1^Department of Pathology, University of Agriculture, Faisalabad, Pakistan; ^2^Institute of Animal Science, Guangdong Academy of Agricultural Sciences, Animal Nutrition and Feed Science in South China, Ministry of Agriculture and Rural Affairs, State Key Laboratory of Livestock and Poultry Breeding, Key Laboratory of Guangdong Public Laboratory of Animal Breeding and Nutrition, Guangdong Key Laboratory of Animal Breeding and Nutrition, Guangzhou, China; ^3^Department of Animal Science & Technology, Shandong Vocational Animal Sciences and Veterinary College, Weifang, China; ^4^Department of Animal Production, Faculty of Agriculture, Benha University, Benha, Egypt; ^5^Department of Parasitology, University of Agriculture, Faisalabad, Pakistan

**Keywords:** IBD, *in-ovo*, vaccine, immunopathology, layers

## Abstract

This study was designed to compare immunopathological effects of *in ovo* vaccination with post-hatch vaccination against IBD in White Leghorn chicks. A total of 189 embryonated eggs were divided into six groups. At day 18 of incubation, groups A–C were administered *in ovo* with 228E, Winterfield 2512:10/3 and 2512/90:10/2.7, respectively, group D (post-hatch vaccination) and group E as shamed control (for quality evaluation of *in ovo* vaccination technique), and group F as control. The results showed that antibody titers against IBD detected by ELISA on days 2, 17, and 28 were significantly higher in all *in ovo* groups as compared to control groups E and F. On day 17, all vaccinated groups (*in ovo* and post-hatch vaccinated) showed no significant differences in antibody titers among themselves; however, at day 28, only the post-hatch group showed significantly higher antibody titers followed by *in ovo* vaccinated groups. The cell-mediated immunity determined by PHA-P assay was significantly higher in all vaccinated groups than the non-vaccinated groups. No clinical signs of IBD infection were observed in any of the vaccinated groups. There was only increase in bursa size of groups vaccinated with intermediate plus strains (groups A, C, and D) at day 28. The histopathology showed that all the treatment groups had mild lesions induced by IBD virus in bursa. This study concluded that *in ovo* vaccination with live IBD vaccines provides protective immunity to the chickens even in the presence of IBD-specific MDA; therefore, the onset of immunity was much earlier than the post-hatch vaccination and *in ovo* groups also maintained protective immunity against IBD for longer time.

## Introduction

Infectious bursal disease virus (IBDV) is a dsRNA virus of birnaviridae family having two serotypes (serotypes I and II), of both only serotype I is responsible for infectious bursal disease (IBD) in chicken also known as “Gumboro diseases.” The virus is difficult to be inactivated by ordinary physical and chemical methods. Therefore, it persists for longer durations in poultry houses. Longer persistence of the diseases in commercial poultry flocks is of prime economic concern that may adversely affect the health and welfare of the flocks (1, 2). Infectious bursal disease is highly contagious in young chicken with acute onset and primarily targets the lymphoid tissue with special affinity to bursa of Fabricius (3, 4). At days 3–4 of post-infection (PI), bursa shows hypertrophy, hyperemia, and edema. At days 5–6 of PI, the bursa regains its normal size and by day 8 gets atrophied to one-third of its normal size. The birds suffer from severe dehydration and exhibit hypertrophy, whitish urate crystal deposits with cell debris in kidneys (5). IBD virus-induced immunosuppression (6) predisposes the young chicks to opportunistic pathogens and prevents the development of adequate immune response to the commonly used vaccines (7). The degree of immunosuppression, mortality rate, clinical signs, and severity of bursal lesions depend upon the age and immune status of the bird and pathogenicity of viral strains (5).

Despite intensive vaccination regimens, IBD still stands as an economically important disease of poultry (8). In field environment, mostly IBD vaccine is administered in drinking water to the chicks. However, *in ovo* vaccine delivery has shown to induce protective immunity as well (9, 10). The concept of *in ovo* vaccination evolved by successful experimental administration of Marek's disease virus (MDV) vaccine to chicken embryos which protected the chicks from MDV challenge later in life (11). Following this successful study, this concept of vaccinating the chicken embryos further developed and the chicken embryos were vaccinated with IBD vaccine alone and in combination with MD vaccine, both of which presented the same conclusion as the previous study (12).

*In ovo* vaccine administration against IBD virus at 18th day of incubation minimizes the susceptibility window, i.e., the duration between the vaccine administration and an early exposure to the IBD virus as compared to routine post-hatch vaccine administration (13). The recent studies have revealed that in comparison with the vaccine administered after hatching, the *in ovo* vaccination approach stimulated both the innate and adaptive immunity in young chicks (13). The IBD virus exposure induced systemic humoral immunity as well as the cell-mediated immune (CMI) response (14). The local immune response may also play a remarkable role in building protection against IBD virus challenge as the IBD virus *via* gut-associated tissue enters the circulation and then gets distributed to other organs (15).

Although conventional vaccination strategies are still in use but *in ovo* vaccination is also being adopted in many parts of the world (16), however, there is less information about the immunopathological sequelae of administering live vaccines by *in ovo* route as the embryos lack fully developed immune system. Therefore, present experimental study was designed to investigate the pathological changes associated with *in ovo* administration of IBD live vaccines and comparison of immune response elicited by *in ovo* vaccination with that of post-hatch vaccination against IBD followed in field conditions.

## Materials and methods

### Experimental design

A total of 189 fertile hatching eggs from White Leghorn (WLH) layer breeder flock were procured from a commercial hatchery. The eggs were clean and shifted to the setter of disinfected incubator in the hatchery at Department of Poultry Science, University of Agriculture Faisalabad. At day 18 of incubation, eggs were candled and only the viable embryos were selected. The egg shell surfaces of all groups except group F (Control) were disinfected with alcohol and groups A, B, C, and E inoculated *in ovo via* air cell route, using a 2.5-cm 23-gauge needle with individual dose (0.1 ml) of vaccine (17) per egg. Gentamicin sulfate was added at dose rate of 1 mg /ml inoculums (18) to avoid bacterial contamination. The virus particles per dose for group A were (2-3 log10 EID_50_), B (10 ^3^ EID_50_), and C (10 ^2.7^ EID_50_). The eggs of group E served as shamed control and were inoculated with sterilized physiological saline (19). A number of eggs in groups A, B, C, D, E, and F were 33, 32, 32, 32, 30, and 30 eggs, respectively. After that, the eggs were placed in labeled porous bags. These egg-containing bags were then carefully shifted to the hatcher of disinfected incubator till day 21 of incubation. The embryonated eggs of commercial layer were from same batch used for *in ovo* vaccination and post-hatch study.

After hatching, the chicks were shifted to the experimental poultry house at Department of Clinical Medicine and Surgery, University of Agriculture Faisalabad. Birds of each group were kept in separate cages and were provided drinking water and feed *ad libitum*. The hatchlings of the *in ovo* vaccinated groups (A, B, C), group D (used for post-hatch vaccination), shamed control (E), and negative control (F) were used in post-hatch experiment of 28 days. The experimental design of chicks is given in Table 1.

**Table 1:** Experimental design for chicks.

**Groups**	**IBDV strains**	**Route of vaccine**	**Description**
**A**	Nobilis Gumboro 228-E	*In ovo*	No post-hatch vaccination of IBD.
	Intermediate plus strains		
**B**	Winterfield 2512: 10/3	*In ovo*	
	Intermediate strain		
**C**	Winterfield 2512/90: 10/2.7	*In ovo*	
	Intermediate plus strains		
**D**	Winterfield 2512/90: 10/2.7	Eye drop	IBD post-hatch vaccination on days 08 and 16.
	Intermediate plus strains		
**E**	Shamed control	*In ovo*	Physiological saline solution (no vaccination of IBD)
**F**	Control	–	No intervention

The only group D was administered post-hatch IBD vaccine by intraocular route (20) at days 08 and 16 as per field practice. However, all the groups were vaccinated with live vaccines *via* eye drop route against Newcastle disease (ND) and infectious bronchitis (IB) at day 5 of age. The duration of experiment was 28 days (because the susceptibility window for IBD infection in layer chicks is at 3–4 weeks of age (21). A total of six birds from each group were bled at days 2 and 28 for collection of blood and visceral organs for evaluation of different parameters. The blood was collected for serum. The bursal tissues were collected at days 2 and 28 and fixed in 10% neutral buffered formalin for histopathology.

### Parameters studied

The percent hatchability was determined. Clinical signs were recorded two times daily and body weights weekly. On days 2 and 28, necropsy examination was done to explore any gross changes in the organs. The preserved tissues were processed for histopathological studies (22).

To determine **cell-mediated immunity**, the cutaneous lymphoproliferative response to phytohemagglutinin (PHA-P) was assessed *in vivo* at 24, 48, and 72 h as cutaneous basophilic hypersensitivity response = skin thickness in the right foot (PHA-P Ag)—skin thickness in the left foot (control) (23). Humoral immunity was determined using ELISA (Iddex Labs USA). The bursa: body weight index was determined by formula of (24):


$$\begin{array}{l} \text{ BW ratio}=\frac{\text{Bursal weight}}{\text{Body weight}}\times 100\\ \text{Bursa}:\text{ BW index}=\frac{\text{Bursa}:\text{ Body weight of vaccinated group}}{\text{Mean bursa}:\text{ Body weight of control}} \end{array}$$

Organ weights of immune organs (bursa, thymus, and spleen) were studied by taking average of six replicates, as absolute organ weights and relative organ weights. The formula for relative organ weight was:


$$\begin{array}{l} \text{Relative organ weight}=\frac{\text{Absolute organ weight}}{\text{Body weight}} \end{array}$$

The data obtained were subjected to one-way analysis of variance and group means were compared by Duncan's multiple range test (DMR) (*p* ≤ 0.05) using M Stat-C software package. All the experimental protocols and use of animal were approved by Graduate Studies Research Board (GSRB) of University of Agriculture Faisalabad.

## Results

### Hatchability

The percent hatchability varied among different groups. The highest percent hatchability was seen in group E followed by groups D, F, A, B, and C. It has been presented in Table 2.

**Table 2:** Hatchability in different groups of the White Leghorn chicks administered live IBD vaccines *in ovo* and post-hatch period.

**Groups**	**Total no. of fertile hatching eggs**	**Hatchability #**	**Hatchability (%)**
A	33	31	93.93
B	32	30	93.75
C	32	30	93.75
D	32	31	96.86
E	30	30	100
F	30	29	96.67

### Clinical signs

During the whole period of study, none of the birds from any group presented any clinical sign of disease based upon the parameters of their alertness, hydration status, and fecal consistency.

### Body weights

Body weights determined at weekly intervals of different groups have been presented in Table 3. No significant difference was found in the weekly body weights among all groups.

**Table 3:** Body weights (mean ± SD) of the White Leghorn chicks administered live IBD vaccines *in ovo* and post-hatch period.

**Groups**	**Vaccine**	**Route**	**Body weight (mean ± SD)**			
			**Week 1**	**Week 2**	**Week 3**	**Week 4**
A	Nobilis Gumboro 228-E	*In ovo*	38.89 ± 1.90	59.13 ± 2.55	107.28 ± 6.34	160.55 ± 3.43
	Intermediate plus strains					
B	Winterfield 2512: 10/3	In ovo	39.45 ± 2.66	60.96 ± 3.32	99.67 ± 7.23	162.87 ± 5.54
	Intermediate strain					
C	Winterfield 2512/90: 10/2.7	*In ovo*	41.62 ± 3.09	60.65 ± 3.64	102.27 ± 10.67	158.14 ± 12.17
	Intermediate plus strains					
D	Winterfield 2512/90: 10/2.7	Eye drop	40.48 ± 4.10	58.0 ± 5.60	106.19 ± 10.09	159.27 ± 12.15
	Intermediate plus strains					
E	Shamed control	*In ovo*	39.39 ± 3.28	59.82 ± 3.55	104.85 ± 13.12	160.15 ± 9.46
F	Control		39.87 ± 4.74	57.83 ± 6.17	106.45 ± 10.19	159.86 ± 11.88

Values denoted by different alphabets in each column are significantly different (p ≤ 0.05).

### Gross lesions

**Figure 1:**
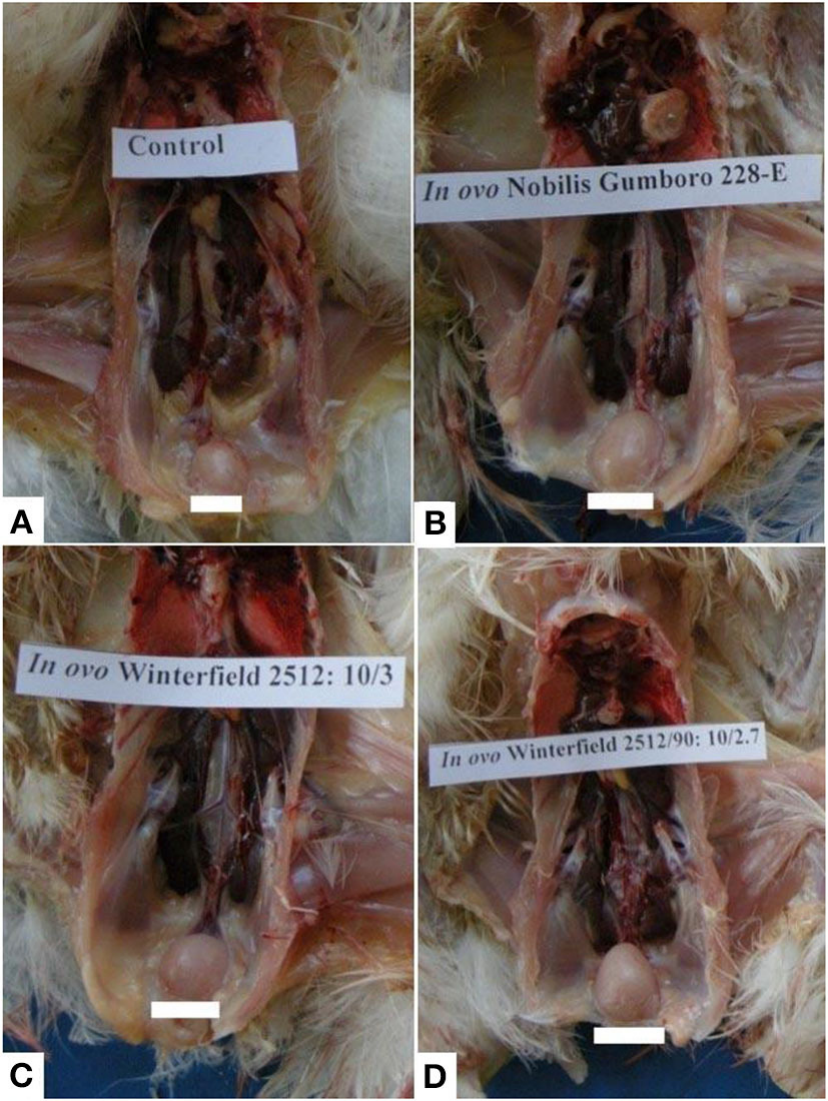
Bursa of Fabricius of layer chicks at day 28. **(A)** Control group (normal size of bursa) whereas bursal hypertrophy **(B–D)** in group A (*in ovo* 228 E), group B (*in ovo* Winterfield 2512: 10/3) and group C (*in ovo* Winter field 2512: 10/2.7) in comparison with control group F.

At day 2 after hatching, all organs including bursa of Fabricius, spleen, kidney, and thymus grossly appeared normal in all groups. At day 28 after hatching, groups E and F showed the normal size of the bursa. However, the bursa of groups A, C, and D was swollen two times their original size followed by those of group B which presented moderate swelling (Figure 1). Other organs such as spleen, kidney, and thymus presented the normal gross appearance.

### Histopathological lesions

#### Bursa of Fabricius

##### Group A (in ovo Nobilis Gumboro 228 E)

**Figure 2:**
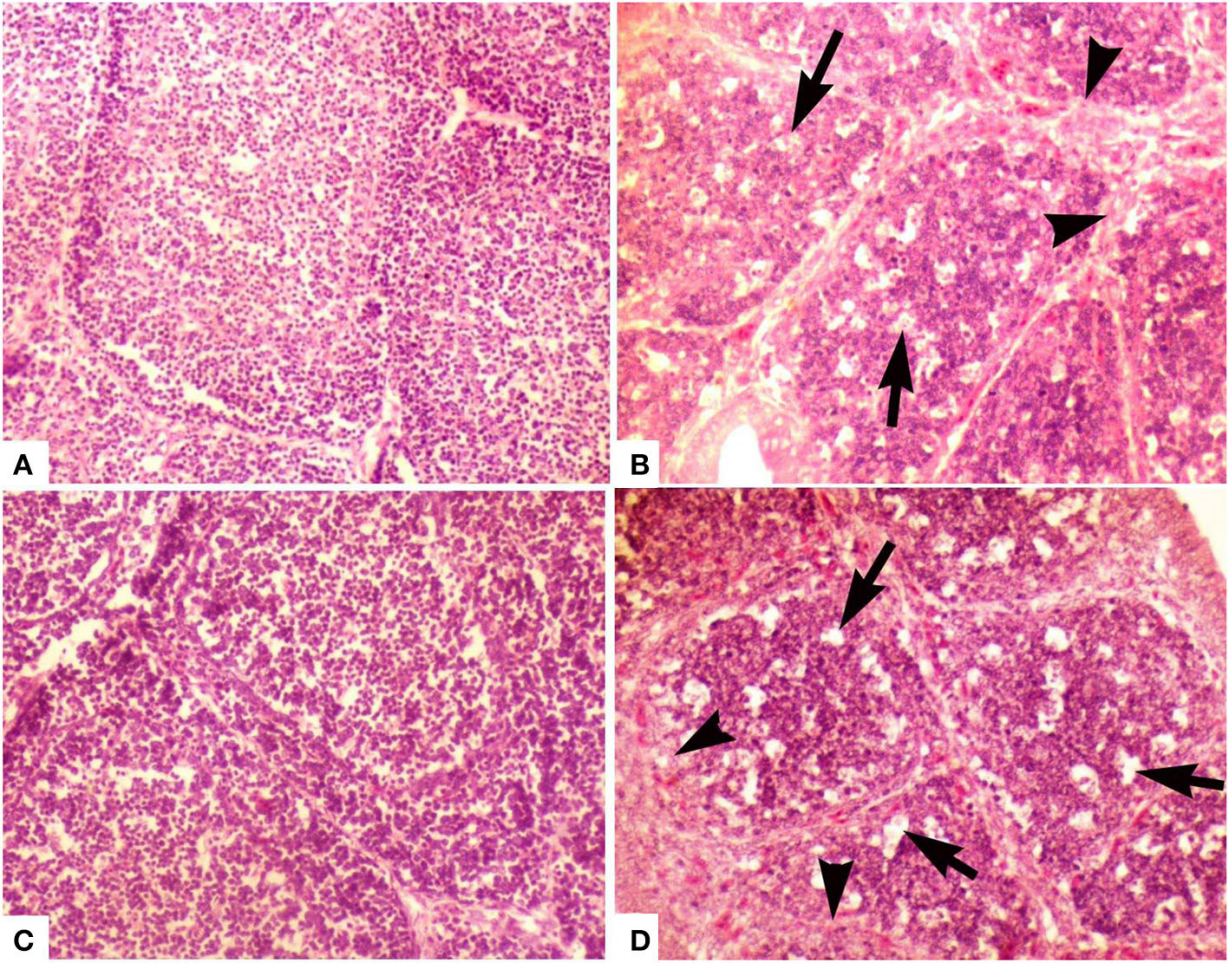
Photomicrograph of bursa of Fabricius of layer chicken on day 28. **(A)** Developed cortex and medulla (post-hatch vaccination group D), **(B)** (group A *in ovo* 228 E) and **(C)** showing demarcated cortex and medulla (group B) (*in ovo* Winterfield 2512: 10/3) (H&E staining at 200×) **(D)** (group C *in ovo* Winterfield 2512: 10/2.7) showing vacuolar degeneration (arrows), polymorphic cells in the medullary region and interfollicular connective tissue fibrosis (arrow heads).

At day 2, the medulla of follicles exhibited mild degree of lymphocytic depletion. However, the surface epithelium was intact. There were prominent dark condensed nuclei (pyknotic) depicting the necrosis in few follicles. A thin layer of cells was present between cortex and medulla. Follicles appeared larger in size compared with follicles of other groups. At day 28, the interfollicular connective tissue was minimal. Medulla was not as densely packed as cortex, and it mainly contained small cells with scarce cytoplasm, some larger cells (macrophages), and fibroblasts. Cortex was densely packed with small and large cells with little cytoplasm. Medullae of bursal follicles also showed some scattered cells with pyknotic nuclei. The cortex contained some empty spaces (Figure 2B).

##### Group B (in ovo Winterfield 2512: 10/3)

At day 2, the empty spaces and pyknotic nuclei in the medulla of follicles appeared lesser in number as compared to groups A and E while at day 28, surface epithelium was intact and interfollicular connective tissue was thinner than group A. There was clearly demarcated cortex and medulla and the cortical cells were larger than medullary cells. Both cortex and medulla were not as densely packed as that of group A. Some cells with pyknotic nuclei and large cells (macrophages) were present in medulla. Most of the cells present in medulla were small with thin rim of cytoplasm. In medulla, lesser fibroblasts and at some places, segmented cells and macrophages were seen more frequently than in group A (Figure 2C).

##### Group C (in ovo Winterfield 2512: 10/2.7)

At day 2, follicular size was variable being smaller in some parts of the bursal tissue while normal in other parts. Lesser pyknotic cells were observed as compared to group A suggesting necrosis in a few follicles. Empty spaces were seen in the follicles; however, the surface epithelium of the organ was found intact. Similar pattern was present day 28 (Figure 2D).

##### Group D (post-hatch Winterfield 2512/90: 10/2.7 eye drop vaccination)

At day 2, empty spaces in the medulla of the follicles were minimum compared with other groups. Medulla and cortex almost indistinguishably separated from each other by a very fine layer of cortical rim around the medulla. The connective tissue was lesser than that observed in group E. The surface epithelium of the bursa of Fabricius was found intact. The interfollicular connective tissue was thin even at day 28. There has been a clear demarcation of cortex and medulla. Medulla was denser than those of groups A, B, and C. Large cells (macrophages) were also present in the medullae; however, there was less fibroblast activity in medulla as compared to groups A and C (Figure 2A).

##### Group E (in ovo shamed control)

Some empty spaces were observed in the medullae of follicles. Medulla and cortex were almost indistinguishable, and a very fine layer of cortical rim formed around the medulla. The connective tissue was thinner than that observed in group F. The surface epithelium of the organ was intact. There was clearly demarcated cortex and medulla. Cortex and medulla were denser than the groups A, B, C, and D. In medulla, most of the cells were small with thin rim of visible cytoplasm, and some macrophages and fibroblasts were also observed.

##### Group F (control)

At day 2, medullary portion of bursal follicles exhibited least empty spaces. Interfollicular connective tissue was prominent. The epithelial folds on the surface of the organ were intact. All follicles showed larger medulla and a thin rim of cortical cells was surrounding it. At day 28, surface epithelium was intact. There was clearly demarcated cortex and medulla. Cortex and medulla were denser than those of groups A, B, C, and D. In medulla, most of the cells were small with thin rim of visible cytoplasm, and some macrophages and fibroblasts were also observed.

##### Cell-mediated immunity

Lymphoproliferative response to PHA-P as elicited by the thickness of skin at the site of injection has been presented in Table 4. At day 8 of age, it was significantly higher in groups A and C compared with control 24 h post-injection. The response was significantly lower in group B compared with groups A and C. The significantly lowest response was observed in groups D, E, and F. At 48 h post-injection, group B showed significantly lower response and all other groups showed similar trend as it was at 24 h. At 72 h post-injection, significantly higher response was observed in group C followed by groups A and B. At day 21 of age, response to PHA-P at 24 and 48 h post-injection was significantly higher in group D whereas groups A, B, and C showed significantly lower response from group D. The groups E and F showed significantly lower values than all other groups. At 72 h post-injection, significantly higher response was observed in group D followed by group B. The groups A and C showed significantly lower response from group B, whereas the groups E and F showed significantly lower response from all other groups.

**Table 4:** Lymphoproliferative response against PHA-P in White Leghorn chicks administered IBD live vaccines by *in ovo* and post-hatch (mean ± SD).

**Group**	**PHA-P response (Skin thickness in mm)**		
	**24 h**	**48 h**	**72 h**
**Day 8**			
A	0.88 ± 0.07 a	0.83 ± 0.06 ab	0.75 ± 0.02 b
Nobilis Gumboro 228-E			
Intermediate plus strains			
(*In ovo* vaccination)			
B	0.82 ± 0.03 b	0.79 ± 0.03 b	0.76 ± 0.02 b
Winterfield 2512: 10/3			
Intermediate strain			
(*In ovo* vaccination)			
C	0.90 ± 0.06 a	0.87 ± 0.07 a	0.83 ± 0.09 a
Winterfield 2512/90: 10/2.7			
Intermediate plus strains			
(*In ovo* vaccination)			
D	0.08 ± 0.04 c	0.06 ± 0.03 c	0.04 ± 0.02 c
Winterfield 2512/90: 10/2.7			
Intermediate plus strains			
(Post-hatch vaccination)			
E	0.08 ± 0.04 c	0.07 ± 0.04 c	0.05 ± 0.03 c
Shamed control			
F	0.09 ± 0.03 c	0.06 ± 0.02 c	0.04 ± 0.02 c
Control			
**Day 21**			
A	0.56 ± 0.02 b	0.51 ± 0.03 b	0.44 ± 0.03 c
(228E)			
B	0.57 ± 0.04 b	0.53 ± 0.05 b	0.50 ± 0.05 b
Winterfield 2512: 10/3			
C	0.56 ± 0.02 b	0.52 ± 0.02 b	0.45 ± 0.03 c
Winterfield 2512/90: 10/2.7			
D	0.77 ± 0.04 a	0.67 ± 0.03 a	0.59 ± 0.05 a
Post-hatch vaccination			
E	0.07 ± 0.01 c	0.04 ± 0.01 c	0.03 ± 0.01 d
Shamed control			
F	0.08 ± 0.02 c	0.05 ± 0.02 c	0.03 ± 0.01 d
Control			

Values denoted by different alphabets in each column are significantly different (p ≤ 0.05).

##### Humoral immunity

At day 2 of age, the White Leghorn (WLH) chicks showed significantly highest ELISA log_2_ titers in groups A, B, and C while significantly lower in groups D, E, and F. At day 17 of age, the ELISA titers were significantly highest in groups A, B, C, and D whereas significantly lower titers were observed in groups E and F. At day 28 of age, significantly higher titers were observed in group D followed by groups A, B, and C compared with control (Table 5).

**Table 5:** ELISA log_2_ titers of the White Leghorn chicks administered live IBD vaccines *in ovo* and post-hatch (means ± SD).

**Groups**	**ELISA log**_2_ **Titers**		
	**Day 2**	**Day 17**	**Day 28**
A	10.98 ± 0.33 a	9.39 ± 0.13 a	8.06 ± 0.80 b
228E			
B	10.97 ± 0.31 a	8.66 ± 0.70 a	7.94 ± 1.08 b
Winterfield 2512: 10/3			
C	10.86 ± 0.09 a	9.33 ± 0.75 a	8.36 ± 0.66 b
Winterfield 2512/90: 10/2.7			
D	9.76 ± 0.224 b	8.83 ± 0.83 a	9.54 ± 0.61 a
Post-hatch vaccination			
E	9.93 ± 0.31 b	6.65 ± 0.48 b	6.95 ± 1.63 cd
Shamed			
F	9.85 ± 0.32 b	6.54 ± 0.79 b	6.74 ± 0.58 d
Control			

Values denoted by different alphabets in each column are significantly different (p ≤ 0.05).

##### Absolute organ weights

The results of absolute organ weights results have been presented in Table 6. At day 2 of age, the White Leghorn chicks of groups A and C showed significantly highest absolute weight of bursa, followed by group B. The significantly lowest bursal weight was observed in groups D, E, and F. The thymus weight was significantly higher in groups A, B, and C whereas significantly lowest thymus weight was observed in groups D, E, and F. The absolute weight of spleen was significantly highest in groups A, B, and C followed by group E and significantly lowest was in groups D and E. At day 28, significantly highest weight of bursa was observed in group D, followed by groups A, B, and C. The significantly lowest bursal weights were observed in groups E and F. The absolute thymic weights were the highest in group D followed by groups B and C which were succeeded by group A. The significantly lowest thymic weight was observed in groups E and F. The absolute weight of spleen was significantly highest in group D followed by groups A, B, and C. The significantly lowest weight of spleen was observed in groups E and F.

**Table 6:** Absolute organ weights (mean ± SD) of the White Leghorn chicks administered live IBD vaccines *in ovo* and post-hatch.

**Groups**	**Bursa of Fabricius**	**Thymus**	**Spleen**
**Day 2**			
A	0.09 ± 0.01 ab	0.08 ± 0.01 a	0.041 ± 0.00 ab
B	0.08 ± 0.01 b	0.07 ± 0.01 a	0.043 ± 0.00 a
C	0.10 ± 0.01 a	0.08 ± 0.00 a	0.048 ± 0.01 a
D	0.03 ± 0.001 c	0.039 ± 0.001 b	0.026 ± 0.001 c
E	0.02 ± 0.00 c	0.04 ± 0.01 b	0.031 ± 0.00 bc
F	0.02 ± 0.00 c	0.04 ± 0.01 b	0.023 ± 0.00 c
**Day 10**			
A	0.24 ± 0.01 a	0.17 ± 0.02 ab	0.11 ± 0.02 a
B	0.21 ± 0.02 b	0.13 ± 0.02 bc	0.09 ± 0.02 b
C	0.25 ± 0.02 a	0.18 ± 0.02 a	0.06 ± 0.01 c
D	0.167 ± 0.03 c	0.120 ± 0.05 c	0.062 ± 0.01 c
E	0.15 ± 0.02 d	0.10 ± 0.01 c	0.09 ± 0.02 b
F	0.14 ± 0.02 e	0.09 ± 0.01 c	0.06 ± 0.01 c
**Day 17**			
A	0.57 ± 0.04 a	0.27 ± 0.02 a	0.15 ± 0.01 a
B	0.38 ± 0.03 b	0.24 ± 0.03a	0.12 ± 0.04 bc
C	0.53 ± 0.05 a	0.26 ± 0.03 a	0.14 ± 0.03 ab
D	0.58 ± 0.06 a	0.27 ± 0.04 a	0.15 ± 0.03 a
E	0.38 ± 0.02 b	0.19 ± 0.01 b	0.10 ± 0.01 bc
F	0.35 ± 0.03 b	0.18 ± 0.02b	0.09 ± 0.02 c
**Day 28**			
A	0.83 ± 0.04 b	0.87 ± 0.07 c	0.33 ± 0.02 b
B	0.83 ± 0.03 b	1.02 ± 0.04 b	0.34 ± 0.02 b
C	0.84 ± 0.09 b	0.98 ± 0.06 b	0.33 ± 0.02 b
D	1.10 ± 0.11 a	1.73 ± 0.05a	0.40 ± 0.06 a
E	0.603 ± 0.02 c	0.613 ± 0.07 d	0.273 ± 0.03 c
F	0.557 ± 0.03 c	0.60 ± 0.08 d	0.24 ± 0.02 c

Values denoted by different alphabets in each column are significantly different (p ≤ 0.05).

##### Relative organ weight

The results of relative organ weights results have been presented in Table 7. The relative organ weights of WL chicks calculated at days 2, 10, and 17 of age showed that the relative weight of bursa and thymus was significantly higher in groups A and C followed by group B which was succeeded by significantly lowest groups D, E, and F. The relative weight of spleen was significantly the highest in groups A, B, and C compared with F. At day 28, the relative weight of bursa and thymus was significantly highest in group D, followed by groups A, B, and C in comparison with E and F. The relative weight of thymus was significantly the higher in group D, followed by groups B and C compared with groups E and F. The relative weight of spleen was significantly highest in group A followed by groups A, B, and C, which were succeeded by group E. The significantly lower relative splenic weights were observed in group F.

**Table 7:** Relative organ weights (mean ± SD) of the White Leghorn chicks administered live IBD vaccines *in ovo* and post-hatch.

**Groups**	**Bursa of Fabricius**	**Thymus**	**Spleen**
**Day 2**			
A	0.23 ± 0.01 a	0.20 ± 0.02 a	0.11 ± 0.01 a
B	0.20 ± 0.01 b	0.18 ± 0.02 b	0.11 ± 0.02 a
C	0.24 ± 0.01 a	0.20 ± 0.01 a	0.12 ± 0.02 a
D	0.065 ± 0.01 c	0.096 ± 0.01 c	0.066 ± 0.02 c
E	0.06 ± 0.01 c	0.09 ± 0.01 c	0.08 ± 0.01 b
F	0.06 ± 0.01 c	0.09 ± 0.01 c	0.06 ± 0.02 c
**Day 10**			
A	0.39 ± 0.02 a	0.29 ± 0.04 a	0.18 ± 0.04 a
B	0.34 ± 0.01 b	0.22 ± 0.03 b	0.14 ± 0.03 ab
C	0.40 ± 0.01 a	0.30 ± 0.05 a	0.10 ± 0.03 c
D	0.290 ± 0.06 c	0.205 ± 0.07 b	0.107 ± 0.02 bc
E	0.25 ± 0.02 d	0.17 ± 0.02 b	0.15 ± 0.03 a
F	0.24 ± 0.02 d	0.17 ± 0.01 b	0.10 ± 0.01 c
**Day 17**			
A	0.53 ± 0.04 a	0.25 ± 0.03 a	0.14 ± 0.02 a
B	0.38 ± 0.01 b	0.24 ± 0.03 a	0.12 ± 0.04 ab
C	0.52 ± 0.01 a	0.25 ± 0.03 a	0.14 ± 0.03 a
D	0.55 ± 0.05 a	0.26 ± 0.05 a	0.14 ± 0.03 a
E	0.363 ± 0.05 bc	0.186 ± 0.02 b	0.098 ± 0.01 bc
F	0.33 ± 0.02 c	0.17 ± 0.01 b	0.09 ± 0.02 c
**Day 28**			
A	0.52 ± 0.02 b	0.54 ± 0.05 c	0.21 ± 0.01 b
B	0.51 ± 0.02 b	0.63 ± 0.03 b	0.21 ± 0.01 b
C	0.53 ± 0.05 b	0.63 ± 0.08 b	0.21 ± 0.02 b
D	0.69 ± 0.03 a	1.08 ± 0.08 a	0.25 ± 0.03 a
E	0.38 ± 0.03 c	0.38 ± 0.06 d	0.17 ± 0.02 c
F	0.36 ± 0.02 c	0.38 ± 0.07 d	0.15 ± 0.01 d

Values denoted by different alphabets in each column are significantly different (p ≤ 0.05).

##### Bursa: Body weight index (BB index)

The results are presented in Table 8. The BB index of WL chicks at day 2 of age was significantly highest in groups A and C followed by group B whereas significantly lowest BB index was recorded in group D. At day 10 of age, BB index was significantly highest in groups A and C succeeded by group B followed by group E while significantly lowest BB index was recorded in group D. At day 17 of age, groups B and E showed significantly lower BB index whereas other groups showed significantly higher BB index which was non-significantly different among themselves. At day 28, group D showed significantly highest BB index followed by groups A, B, and C whereas group E showed significantly lowest BB index.

**Table 8:** Bursa: Body weight index of the White Leghorn chicks administered live IBD vaccines *in ovo* and post-hatch period at days 2, 10, 17, and 28.

**Groups**	**Bursa: body weight index**			
	**Day 2**	**Day 10**	**Day 17**	**Day 28**
A (228E)	4.003 ± 0.21 a	1.656 ± 0.07 a	1.612 ± 0.11 a	1.441 ± 0.04 b
B (Winterfield 2512: 10/3)	3.542 ± 0.22 b	1.411 ± 0.06 b	1.160 ± 0.04 b	1.416 ± 0.04 b
C (Winterfield 2512/90: 10/2.7)	4.201 ± 0.26 a	1.681 ± 0.06 a	1.582 ± 0.03 a	1.481 ± 0.14 b
D (Post-hatch vaccination)	1.14 ± 0.14 c	1.21 ± 0.224 d	1.676 ± 0.15 a	1.907 ± 0.09 a
E (Shamed)	1.000 ± 0.09 c	1.053 ± 0.08 c	1.10 ± 0.152 b	1.05 ± 0.08 c

Values denoted by different alphabets in each column are significantly different (p ≤ 0.05).

##### Histomorphometry of bursa of Fabricius

The histomorphometry of bursa of Fabricius at 2 day of age of birds showed significantly highest follicular diameter in group B, followed by groups D, E, and F, whereas significantly lowest follicular diameter was observed in groups A and C. The cortex diameter was significantly higher in group B and C compared with control group F. However, there was a non-significant difference among groups B and D, E, and F. The diameter of medulla was significantly higher in groups B and D, succeeded by groups E and F. The interfollicular tissue was significantly highest in groups A, C, D, and E as compared to control whereas significantly lower interfollicular connective tissue was observed in group B compared with control (Table 9).

**Table 9:** Histomorphometery of bursa of Fabricius of the White Leghorn chicks administered live IBD vaccines *in ovo* and post-hatch period at days 2 and 28 of age.

**Groups**	**Follicle diameter μm (mean ± SD)**	**Cortex diameter μm (mean ± SD)**	**Medulla diameter μm (mean ± SD)**	**Interfollicular connective tissue μm (mean ± SD)**
**Day 2**				
A (228E)	99.62 ± 11.02c	10.03 ± 2.22b	79.55 ± 9.32c	10.03 ± 2.22ab
B (Winterfield 2512: 10/3)	144.77 ± 18.38a	12.18 ± 3.24ab	120.40 ± 12.16a	7.17 ± 3.51b
C (Winterfield 2512/90: 10/2.7)	100.33 ± 12.06c	15.05 ± 3.60a	70.23 ± 12.36c	11.47 ± 2.22a
D (Post-hatch vaccination)	126.85 ± 23.35b	10.03 ± 2.22b	106.78 ± 24.69ab	9.32 ± 3.24ab
E (Shamed)	123.27 ± 7.02b	11.47 ± 2.22b	100.33 ± 7.02b	7.88 ± 3.24ab
F (Control)	118.97 ± 12.66b	10.03 ± 2.22b	98.90 ± 9.02b	11.47 ± 2.22a
**Day 28**				
A (228E)	342.57 ± 46.29a	50.88 ± 20.43a	240.80 ± 21.07a	17.20 ± 3.85a
B (Winterfield 2512: 10/3)	255.13 ± 11.75b	31.53 ± 7.02ab	192.07 ± 16.01b	15.77 ± 3.51a
C (Winterfield 2512/90: 10/2.7)	229.33 ± 16.91b	25.80 ± 6.08b	177.73 ± 7.02b	16.48 ± 3.24a
D (Post-hatch Vaccination)	326.80 ± 69.87a	48.02 ± 17.07a	230.77 ± 44.66a	16.48 ± 4.23a
E (Shamed)	258 ± 39.60b	41.57 ± 18.78ab	174.87 ± 19.36b	16.48 ± 5.03a
F (Control)	260.15 ± 54.51b	42.28 ± 22.33ab	175.58 ± 21.49b	15.05 ± 3.60a

Values denoted by different alphabets in each column are significantly different (p ≤ 0.05).

At day 28, the follicular diameter was significantly higher in groups A and D whereas it was compared with control group F. The diameter of the cortex was significantly higher in groups A, B, D, and E as compared to control group whereas it was significantly lower in group C. The diameter of medulla was significantly higher in groups A and D in comparison with group F. The interfollicular connective tissue showed no significant difference among all groups.

## Discussion

Infectious bursal disease is a highly contagious and immunosuppressive disease of economic importance for commercial poultry (8). Chickens of all breeds are susceptible to IBD; however, White Leghorn shows higher morbidity and mortality rate. Vaccination is the principle control strategy for IBD (3) and contributes to minimize the IBD-related losses. However, there is no vaccine and vaccination technique which could provide 100% protection. Hence, a rational and economically viable control strategy is required to culminate the disease (6).

*In ovo* vaccination of chick embryos is among contemporary vaccination strategies. The concept of this vaccination protocol is to stimulate the developing immune system in late embryonic life against probable IBDV challenge in post-hatch life. The *in ovo* vaccination reduces the cost of labor and can initiate primary immune response even in the presence of maternal antibodies (7, 25). Some studies suggested that there are few live vaccines used in post-hatch vaccination regimes which can be considered for *in ovo* administration without any detrimental effects on the survival of chicken embryos (26).

The results of this study revealed that all groups administered physiological saline or live vaccines *via in ovo* route produced hatchability percentage of 90.91, 93.75, 90.63, and 100% in groups A (intermediate plus strain; Nobilis Gumboro 228 E), B (intermediate strain; Winterfield 2512: 10/3), C (intermediate plus strain; Winterfield 2512/90:10/2.7), and E (shamed; physiological saline), respectively. This finding indicates that neither *in ovo* administration technique nor the post-hatch delivery of live IBD vaccines negatively affected the hatchability percentage or the survival of newly hatched chicks. Our findings are in line with the suggestions of Romao et al. (19) and Saeed et al. (27). However, the hatchability percentage in this experiment was higher than that of commercial hatcheries because all non-viable eggs were discarded at day 18 of incubation after candling as suggested by Moura et al. (28).

None of the vaccine treatment groups irrespective of vaccination method (whether *in ovo* or post-hatch vaccination) showed clinical presentation of disease. The results suggested that the birds, of which their embryos subjected to *in ovo* vaccination, did not undergo much stress to yield post-hatch clinical IBD. Similarly, Hedayati et al. (29) and De Wit et al. (25) reported no clinical signs or ailment in chicks administered *in ovo* IBD vaccines.

The body weights of the layer type chick vaccinated (*in ovo* or post-hatch vaccinated) with intermediate vaccinal strain (Winterfield 2512: 10/3), and intermediate plus strains (Nobilis Gumboro 228 E and Winterfield 2512/90:10/2.7) showed no significant differences from the control negative groups. Otsyina et al. (30) demonstrated no deleterious effect of intermediate plus vaccination of White Leghorn chicks upon body weights. Similarly, Ashash et al. (7) and De Wit et al. (25) have shown that *in ovo* vaccination against IBD with live vaccines does not interfere with the body weights of the chickens.

The absolute and relative bursal weight in this study was significantly higher in chicks administered *in ovo* intermediate plus strains (Nobilis Gumboro 228 E and Winterfield 2512/90:10/2.7) than all other groups at day 2 and 10. On day 17, the highest bursal weight was recorded in chicks administered *in ovo* and intermediate plus strains (Nobilis Gumboro 228 E and Winterfield 2512/90:10/2.7) and the post-hatch vaccinated group. On day 28, the significantly highest bursal weight was recorded in chicks of post-hatch vaccinated group. These findings were contrary to the findings of Rautenschlein et al. (31) who reported a decrease in bursal weight of broiler chicks administered intermediate plus vaccine. A similar trend was observed in the relative weight of the broiler chicks administered *in ovo* intermediate plus vaccines. Different authors have shown decreased bursal weights in White Leghorn chicks administered intermediate plus vaccines (30). An absence of decrease in bursal weight of chicks administered intermediate plus vaccine by *in ovo* technique indicated that the negative effect of intermediate plus vaccine upon bursal weight was prevented and it could be helpful in the prevention of possible immunosuppression caused by these stronger vaccines.

The bursa showed minor histopathological changes in *in ovo* vaccinated groups. Similarly, the bursa of post-hatch vaccinated group showed mild lesions as investigated by Rautenschlein et al. (31) and Giambrone et al. (32).

The humoral immunity determined by ELISA showed that the IBD-specific antibody titers were significantly higher for *in ovo* vaccinated groups than control and shamed group at day 2 post-hatching, and similar observations have been reported by Corley et al. (9) and Coletti et al. (33), whereas at day 17, the antibody titers of all vaccinated groups (*in ovo* and post-hatch vaccinated) were showed significantly higher titers than control negative and shammed control groups; however, all vaccinated groups showed non-significant difference in titers among themselves. At day 28, the post-hatch vaccination group showed significantly highest antibody titers. In some recent studies, it has been determined that live vaccines of IBD do not interfere with the maternal antibodies of the commercial chicken in embryonic life or at early post-hatch vaccination; rather, the multiplication of live vaccine virus slows down due to unknown mechanisms (7, 25). Moreover, the mean titers in breeder flock were 9.5 (ELISA log_2_ titers). Despite the higher levels of maternal antibodies transferred to the experimental/ commercial flock, none of the vaccination method of vaccine strain's antibody titers seemed to be affected by breeders' antibody level in this study.

The cell-mediated immune response to PHA-P antigen at day 8 was statistically non-significant between both *in ovo* administered intermediate plus strains but were significantly higher than intermediate strain. While at day 21, the post-hatch vaccinated group showed significantly higher response to PHA-P antigen followed by a non-significant difference among *in ovo* vaccinated groups (irrespective of different levels of virulence of vaccinal strains). However, all vaccinated groups showed significantly higher responses than control group. Sharma et al. (34), however, reported opposite results using the field isolates of IBDV. A reason for the difference in the present result and those of Sharma et al. (34) could be that they used field isolates of IBDV, which might be high in virulence compared to vaccinal strains used in the present study. The purpose of monitoring CMI using PHA-P assay was to assess whether there had been any kind of immunosuppression (in peripheral t-cell activity) in response to live IBD vaccines (especially intermediate and intermediate plus vaccines), which was not observed in this study.

## Conclusion

From the results of this study, it might be concluded that *in ovo* vaccinating approach reduces the susceptibility period of chickens to field IBDV challenge and the birds' immune organs start getting functional even before hatching. In comparison with the post-hatch vaccination strategy, the *in ovo* vaccinated chickens not only develop humoral and cell-mediated immunity much earlier, but also the antibody induction is higher in birds vaccinated *via in ovo* route.

## Data availability statement

The datasets presented in this article are not readily available because not applicable. Requests to access the datasets should be directed to dr.iqzaheer@gmail.com.

## Ethics statement

The animal study was reviewed and approved by Institutional Bioethics Committee, University of Agriculture, Faisalabad.

## Author contributions

IZ, AK, MS, and MK contributed to conception and design of the study. IZ and TZ organized the database. IZ performed the statistical analysis. IZ and MK wrote the first draft of the manuscript. IZ, MS, TZ, AE, and WC wrote sections of the manuscript. WC and AE were partial funding collaborators in this project. All authors contributed to manuscript revision, read, and approved the submitted version.

## Funding

This work was supported by the National Key Research and Development Program (Grant Nos. 2018YFE0128200 and 2021YFD1300405), Fund for China Agricultural Research System (CARS-42-13), Modern Agricultural Industry Technology System Innovation Team of Guangdong Province (2019KJ137), Key Project of the Science and Technology Program of Guangzhou City (Grant No. 2019A050505007), Foreign Expert Project (QNL20200130001), the Science and Technology Program of Guangdong Province (2021A0505050003), Special Fund for Scientific Innovation Strategy-Construction of High Level Academy of Agriculture Science (R2020PY-JX008), and the Science and Technology Program of Guangdong Academy of Agricultural Sciences (202106TD), opening project for State Key Laboratory of Livestock and Poultry Breeding (2021GZ06).

## Conflict of interest

The authors declare that the research was conducted in the absence of any commercial or financial relationships that could be construed as a potential conflict of interest.

## Publisher's note

All claims expressed in this article are solely those of the authors and do not necessarily represent those of their affiliated organizations, or those of the publisher, the editors and the reviewers. Any product that may be evaluated in this article, or claim that may be made by its manufacturer, is not guaranteed or endorsed by the publisher.
